# Identification of Novel Target Genes for Safer and More Specific Control of Root-Knot Nematodes from a Pan-Genome Mining

**DOI:** 10.1371/journal.ppat.1003745

**Published:** 2013-10-31

**Authors:** Etienne G. J. Danchin, Marie-Jeanne Arguel, Amandine Campan-Fournier, Laetitia Perfus-Barbeoch, Marc Magliano, Marie-Noëlle Rosso, Martine Da Rocha, Corinne Da Silva, Nicolas Nottet, Karine Labadie, Julie Guy, François Artiguenave, Pierre Abad

**Affiliations:** 1 INRA, UMR 1355 ISA, Institut Sophia Agrobiotech, Sophia-Antipolis, France; 2 CNRS, UMR 7254 ISA, Institut Sophia Agrobiotech, Sophia-Antipolis, France; 3 Université de Nice Sophia-Antipolis, UMR ISA, Institut Sophia Agrobiotech, Sophia-Antipolis, France; 4 CEA-Institut de Génomique, GENOSCOPE, Centre National de Séquençage, Evry, France; University of Pittsburgh, United States of America

## Abstract

Root-knot nematodes are globally the most aggressive and damaging plant-parasitic nematodes. Chemical nematicides have so far constituted the most efficient control measures against these agricultural pests. Because of their toxicity for the environment and danger for human health, these nematicides have now been banned from use. Consequently, new and more specific control means, safe for the environment and human health, are urgently needed to avoid worldwide proliferation of these devastating plant-parasites. Mining the genomes of root-knot nematodes through an evolutionary and comparative genomics approach, we identified and analyzed 15,952 nematode genes conserved in genomes of plant-damaging species but absent from non target genomes of chordates, plants, annelids, insect pollinators and mollusks. Functional annotation of the corresponding proteins revealed a relative abundance of putative transcription factors in this parasite-specific set compared to whole proteomes of root-knot nematodes. This may point to important and specific regulators of genes involved in parasitism. Because these nematodes are known to secrete effector proteins *in planta*, essential for parasitism, we searched and identified 993 such effector-like proteins absent from non-target species. Aiming at identifying novel targets for the development of future control methods, we biologically tested the effect of inactivation of the corresponding genes through RNA interference. A total of 15 novel effector-like proteins and one putative transcription factor compatible with the design of siRNAs were present as non-redundant genes and had transcriptional support in the model root-knot nematode *Meloidogyne incognita*. Infestation assays with siRNA-treated *M. incognita* on tomato plants showed significant and reproducible reduction of the infestation for 12 of the 16 tested genes compared to control nematodes. These 12 novel genes, showing efficient reduction of parasitism when silenced, constitute promising targets for the development of more specific and safer control means.

## Author Summary

Plant-parasitic nematodes are annually responsible for more than $100 billion crop yield loss worldwide and those considered as causing most of the damages are root-knot nematodes. These nematodes used to be controlled by chemicals that are now banned from use because of their poor specificity and high toxicity for the environment and human health. In the absence of sustainable alternative solutions, new control means, more specifically targeted against these nematodes and safe for the environment are needed. We searched in root-knot nematode genomes, genes conserved in various plant-damaging species while otherwise absent from the genomes of non target species such as those of chordates, plants, annelids, insect pollinators and mollusks. These genes are probably important for plant parasitism and their absence from non-target species make them interesting candidates for the development of more specific and safer control means. Further bioinformatics pruning of this set of genes yielded 16 novel candidates that could be biologically tested. Using RNA interference, we knocked down each of these 16 genes in a root-knot nematode and tested the effect on plant parasitism efficiency. Out of the 16 tested genes, 12 showed a significant and reproducible diminution of infestation when silenced and are thus particularly promising.

## Introduction

Plant-parasitic nematodes (PPN) cause significant damage to agriculture throughout the world. A global survey in 1987 evaluated crop losses at $78–125 billion per year [1]. More recent direct global estimates are not available, but when the increase in agricultural productivity is taken into account, the extrapolated 2001 loss for crops totaled $118 billion (11% of production) [2]. The current figure is thus probably much higher. Measures such as growing resistant crop varieties and the use of nematicides are extensively employed to control PPN infections. Billions of Euros have been spent annually on soil fumigants and other nematicides. Current and previous chemical controls against nematodes are not only costly but they are highly toxic and hazardous, and involve application of environmentally unacceptable compounds. Such toxicological problems and environmental damage caused by nematicides have led to banning of the most efficient chemicals that were commonly used so far (EC directive 2007/619/EC). In the absence of alternative control methods or development of specific and environmentally safe molecules, severe crop losses within major sectors of the agricultural industry are a distinct possibility. Indeed, nematode problems recently re-emerged in some areas where the use of traditional nematicides had been abandoned for a short while [3], [4]. Therefore, novel control measures are urgently needed. The identification of PPN-specific genes expressed during the interaction with the plant host is one of the most promising approaches for identification of new anti-parasitic strategies.

Infective PPN larvae in the soil are nearly microscopic worms, virtually invisible to the naked eye. Although a few nematode species feed on above ground plant parts, such as leaves, stems, flowers, and seeds, the majority of these parasites feed on underground parts of plants, including roots, bulbs, and tubers. Most PPN feed on root tissue and damage their host mainly by stunting the root system, resulting in reduced water uptake and by promoting microbial infections through wound sites or by serving as vectors for pathogenic viruses. Some nematode species exhibit a hit-and-run strategy, remaining migratory during their plant root-associated life cycle. An increase in complexity of host-parasite interactions is observed in sedentary parasite species with their enhanced capacity to manipulate host plant genes in their favor [5]. These endoparasitic nematodes settle down after an initial migratory phase and assume a sedentary life style while transforming plant cells into complex feeding structures. Nematodes of this category represent the most damaging species for crops. Some of these nematodes have a relatively specialized host range (e.g. cyst nematodes *Heterodera* and *Globodera* genus) while others are able to reproduce on thousands of unrelated host plant species (e.g. root-knot nematodes, *Meloidogyne* spp.).

Because root-knot nematodes represent the most economically-important PPN, they constitute the most explored group of species and can now be considered as one of the most advanced models for understanding mechanisms of plant parasitism in nematodes. As with other PPN, they have a syringe-like stylet that is used to pierce and penetrate plant cell walls, to release esophageal secretions into the host tissue and to take up nutrients. During their infective life-cycle root-knot nematode larvae penetrate plant root tissue and migrate along the vascular cylinder. By injecting secretions into plant cells, they induce the formation of a feeding site indispensable for their development. As a consequence of the formation of these feeding structures, root-knots or galls are observed as symptoms of the infestation. Plant nutrient and water uptake are substantially reduced by the resulting damage to the root system, and infested plants are therefore weak and give low yields. Once the feeding structure is established, female nematodes continue their development and eventually become pear-shaped and produce hundreds to thousands of eggs. These eggs are then extruded as an egg-mass, protected within a gelatinous matrix, at the outer surface of the root.

Mining the genomes of root-knot nematodes [6], [7], [8] through an evolutionary and comparative genomics approach, we searched genes conserved in various plant-damaging species while otherwise absent from the genomes of non target species such as those of chordates, plants, annelids, insect pollinators and mollusks. We identified a set of root-knot nematode genes absent from non-target species but present in several plant-damaging organisms. Further bioinformatics pruning of this set of genes yielded new candidates that were silenced using RNA interference (RNAi). Upon silencing experiments, 75% of the candidates induced a significant and reproducible diminution of infestation and are thus particularly promising for the development of new and more specific control strategies.

## Results

### Elimination of root-knot nematode genes shared by non-parasitic species

**Figure 1:**
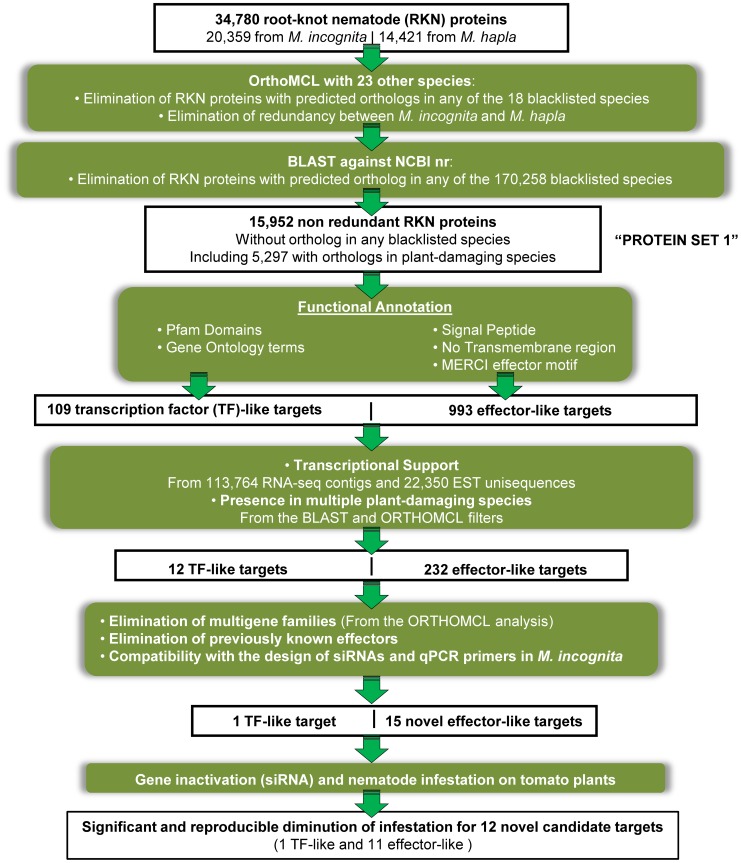
Whole RKN proteomes filtering pipeline for identification of novel targets. Pipeline illustrating the main filtering steps, from the two RKN whole protein sets, that allowed identification of novel and non-redundant targets for the development of specific and safer control methods.

Our main objective was to identify root-knot nematode (RKN) genes that could be used as targets for the development of new control means against these pests. As we absolutely wanted to minimize the risk of collateral effects and preserve non-targeted species, we systematically discarded RKN genes that had putative homologs in non-target species (Figure 1 and methods).

**Figure 2:**
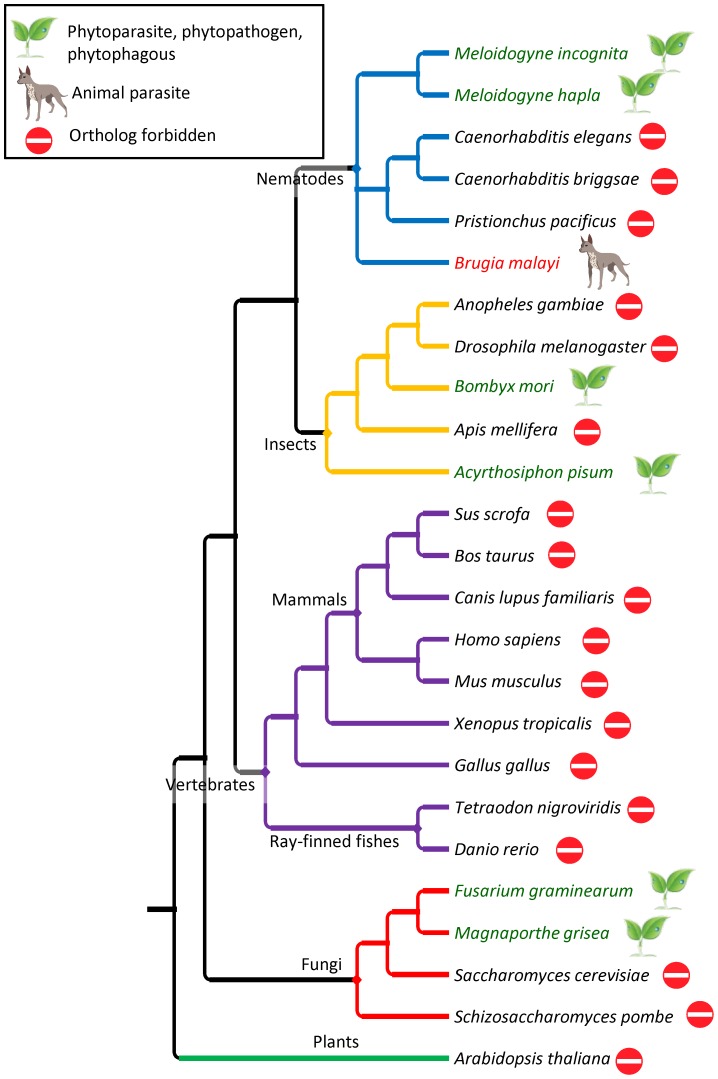
Phylogenetic tree of selected species for OrthoMCL comparison. The relative phylogenetic position and simplified taxonomy of the 25 species included in the OrthoMCL comparison of whole proteomes. The topology is according to the NCBI's taxonomy, except within the nematode and insecta lineages which are according to ref [45] and to ref [46], respectively. Species that are known plant-parasites plant-pathogens or phytophagous are highlighted in green and with a plant symbol. The animal-parasitic nematode *B. malayi* is highlighted in red and with a dog symbol. All the other species, in black, with a “wrong way” sign, are blacklisted.

To select RKN proteins without predicted homologs in non-target species, we first performed an OrthoMCL [9] analysis comparing all predicted proteins in *M. incognita* and *M. hapla* (34,780 proteins) with the whole proteomes of 23 other species (Figure 2). This step was aimed at eliminating RKN proteins having evident orthologs in fully-sequenced non-target genomes and to substantially reduce the number of proteins that will be subsequently compared against the NCBI's nr library. We selected, in priority, species whose whole genomes have been annotated to a quality level allowing a reliable prediction of the ensemble of protein-coding genes. Our selection of species comprised 4 other nematodes, 5 insects, 9 vertebrates (including mammals, ray-finned fishes, amphibian and sauropsida), 4 fungi and 1 plant. Among selected species, we included two plant-pathogenic fungi (*Magnaporthe grisea* and *Fusarium graminearum*), one nematode parasite of animals (*Brugia malayi*) and two insects that feed on living plant tissue (*Acyrthosiphon pisum* and *Bombyx mori*). The 18 other species were blacklisted and whenever a RKN protein had a predicted ortholog in these blacklisted species, the protein was discarded from the rest of the analysis. According to OrthoMCL, a total of 15,181 RKN proteins had a predicted ortholog in at least one blacklisted species and were thus eliminated. The rest of RKN proteins (19,599) had no predicted ortholog in any of the blacklisted species and passed this first filter. Among these proteins, a total of 2,446 were redundant between *M. incognita* and *M. hapla*. To avoid redundancy, and because subsequent biological assays will be performed in *M. incognita*, we kept as representative the *M. incognita* versions. At the end of this first filtering step, a total of 17,153 *Meloidogyne* proteins were kept for further analysis.

Although, with a total of 25 species representing >500,000 proteins, the OrthoMCL analysis we performed is far from negligible, this only represents a limited sample of the whole sequence biodiversity available in public databases. Thus, using a BLASTp [10] analysis, we compared the 17,153 RKN proteins that passed the OrthoMCL filter against the NCBI's nr library. Applying a similar filter as was applied to the OrthoMCL results, we systematically eliminated RKN proteins having putative orthologs in non-target, blacklisted species. Those that had no putative ortholog in any of the blacklisted species or returned no significant similarity at all in any other species, were kept for subsequent analysis. Because there is no comprehensive database indicating the lifestyles of the plethora of species with a sequence in the nr library, we generated a list of blacklisted taxa (methods). In total, our blacklist included 170,258 species covering 4 whole clades (annelida, chordata, mollusca and viridiplantae) in addition to the 18 species already blacklisted in the OrthoMCL analysis. Overall, a total of 10,105 RKN proteins did not return any significant BLASTp hit in nr using the thresholds we had set (methods). More than half of these proteins (5,536) also had no predicted ortholog in the OrthoMCL analysis and were thus considered as potentially orphan or restricted to RKN at this stage. In contrast, 1,201 RKN proteins returned significant BLASTp hits in at least one blacklisted species and were discarded. In total, 15,952 RKN sequences were kept and constituted our protein set 1. This set 1 represents RKN proteins predicted to be absent from blacklisted species and possibly present in other plant-damaging species.

### Conservation of root-knot nematode proteins in plant-damaging species

We assessed whether part of the RKN proteins absent from non-target species were present in other plant-damaging species. The rationale of this analysis is that the more a gene is shared between plant pests while absent from other species, the more it is likely to be involved in core interaction processes with the plant. To assess conservation in plant-damaging species, we filtered the results of both the OrthoMCL and BLASTp analyses. In the OrthoMCL analysis, two plant-pathogenic fungi were included as well as two insects that feed on plant. A total of 4,398 RKN proteins had predicted orthologs in, and only in, these plant-damaging species. Similarly to the list of blacklisted species for the BLASTp filtering, we built up a list of 28,054 potentially plant-damaging species in the NCBI's taxonomy (methods). We identified 1,252 RKN proteins that returned significant BLASTp hits with at least one plant-damaging species. After removing redundancy between the OrthoMCL and BLASTp analyses, we obtained a non-redundant list of 5,297 RKN proteins absent from non-target species but present in at least two plant-damaging species.

### Functional annotation of root-knot nematode proteins and further filtering

To gain functional insight on the proteins that appeared restricted to RKN and other plant-damaging species, we searched and retrieved a series of functional annotations. This included a search for signal peptides for secretion, a search for transmembrane regions, a search for known protein domains and associated functional annotations. We also assessed whether corresponding genes had transcriptional support.

#### Predicted functions of RKN proteins

Out of the 15,952 RKN proteins in set 1 (i.e. that passed both the OrthoMCL and BLASTp filters), only 3,835 or 24% have been assigned a Pfam domain. This is in contrast with the two whole RKN proteomes. Indeed, a total of 10,379 *M. incognita* proteins out of 20,359 [6] and 7,151 *M. hapla* proteins out of 14,421 [7] have been assigned at least one Pfam domain, representing 51% and 49.6% of their respective proteomes (methods).

Echoing the scarcity of Pfam domains assigned to proteins in set 1, only 2,255 proteins (or 13.8%) out of the 15,952 present in set 1 have been assigned a Gene Ontology (GO) term. By comparison, GO terms were assigned to 6,881 (33.8%) and 4,673 (32.4%) of *M. incognita* and *M. hapla* whole proteomes, respectively (methods).

#### Transcription-related proteins were more abundant in RKN-restricted proteins

**Figure 3:**
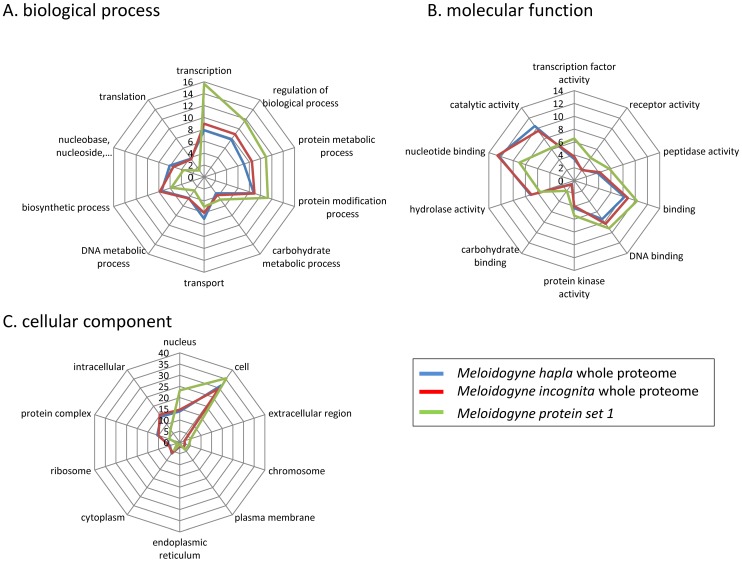
Gene Ontology terms relative abundance in candidate targets compared to whole proteomes. Kiviat diagram representing, the relative abundance of Gene Ontology (GO) terms, in percent for the whole *M. incognita* and *M. hapla* proteomes (in blue and red, respectively) as well as for the proteins that passed the OrthoMCL and BLAST filters (protein set 1 in green). (A) Relative abundance of GO terms assigned to whole RKN proteomes and protein set 1in the Biological Process category. (B) Relative abundance of GO terms assigned to whole RKN proteomes and protein set 1 in the Molecular Function category. (C) Relative abundance of GO terms assigned to whole RKN proteomes and protein set 1 in the Cellular Component category. In the three categories, the ten GO terms that presented the most different relative abundance (in percent) in protein set 1 compared to the whole RKN proteomes are presented.

Using a without *a priori* approach, we searched functional categories present in protein set 1. We compared the relative abundance of the different GO terms between protein set 1 and the two RKN whole proteomes. We remarked that despite substantially different numbers of predicted proteins in *M. incognita* and *M. hapla*, the relative abundance of GO terms was very similar in the two whole proteomes (Table S1, Figure 3). The higher number of protein models in *M. incognita* is due to its bigger genome with a peculiar structure, mainly constituted of regions in two copies with substantial divergence [7].

In contrast, we noticed that, compared to the two RKN whole proteomes, several GO terms were substantially over-represented or under-represented in set 1, for the three different GO ontologies, “biological process”, “molecular function” and “cellular component” (Figure 3). For instance, in the “biological process” ontology, we remarked an over-representation of the term “transcription” in set 1 (∼15.6%) compared to RKN proteomes (8–9%, p-values 6.12E^−17^ - 1.67E^−13^). The term “regulation of biological processes” was also more frequent in set 1 (∼11.7%) compared to the RKN proteomes (8–9%, p-values 5.84E^−6^ - 8.59E^−4^). Conversely, some terms were less frequent in set 1 as compared to the whole RKN protein sets. For example, the term “translation” represented only ∼1.3% of GO terms in set 1, while it represented ∼3.7–3.8% in whole RKN proteomes (p-values 3.21E^−8^ - 3.65E^−8^).

In the “molecular function” ontology, mirroring observations on the “biological process” ontology; we remarked an over-representation of the term “Transcription factor activity” in set 1 (∼6.5%) compared to the whole RKN proteomes (3.3–3.7%, p-values 8.81E^−11^ - 2.53E^−9^). Besides transcription-related terms, we also noticed an over-representation of the terms “receptor activity” (∼4.3% vs. 2.0–2.1%, p-values 6.17E^−11^ - 2.89E^−7^) and “peptidase activity” (∼5.7% vs. 3.8–4.2%, p-values 6.67E^−5^ - 6.69E^−4^).

In the “cellular component” ontology, we noted that the “nucleus” term was over-represented (23.3%) in set 1 compared to whole RKN proteomes (13.8–14.6%, p-values 3.65E^−7^ - 1.38E^−6^). Interestingly, more than half (109) of the 190 proteins annotated as localized in the nucleus in set 1 are also annotated as transcription factors in the “molecular function” ontology. Hence the over-representation of the nucleus localization in set 1 is essentially due to the relative abundance of putative transcription factors.

**Figure 4:**
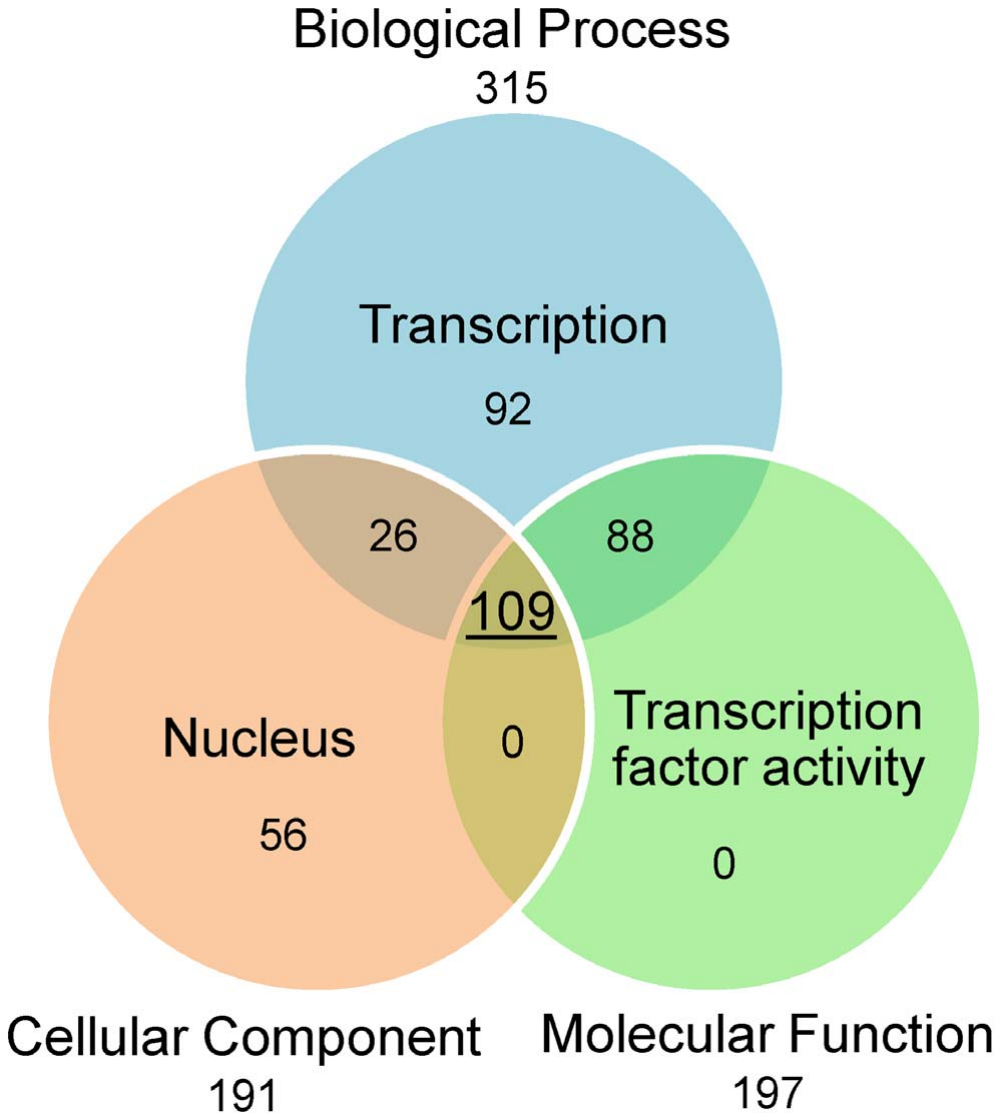
Gene Ontology annotation of putative transcription factors. This Venn diagram represents the number of proteins that cumulate transcription-related terms in their Gene Ontology annotation in protein set 1. A total of 315 proteins have been assigned the term ‘Transcription’ in their Biological Process G.O. annotation. A total of 191 proteins have been assigned the term ‘Nucleus’ in their Cellular Component G.O. annotation. A total of 197 proteins have been assigned the term ‘Transcription factor activity” in their Molecular Function G.O. annotation. Overall, a total of 109 proteins in set 1 cumulate these three transcription factor-related G.O. terms.

Because putative transcription factors specific from RKN and other plant-damaging organisms constitute interesting potential targets, we searched, in set 1, proteins that were annotated with the term “transcription” in the “biological process” ontology, with the term “transcription factor activity” in the “molecular function” ontology and with the term “nucleus” in the “cellular component” ontology. We found a total of 109 RKN proteins that cumulated these three annotations (Figure 4).

### Effector-like proteins specific to phytoparasites

Root-knot nematodes and other plant parasites secrete, into plant tissue, proteins that support successful parasitism. In nematodes, these proteins, called effectors are generally produced in esophageal gland cells and secreted *via* a syringe-like stylet in plant tissue. Several RKN effectors have been characterized so far and shown to support parasitism by playing roles in different key processes such as degradation of the plant cell wall, suppression of plant defense, manipulation of plant cells to produce feeding structures or interaction with plant signaling pathways [11], [12], [13]. Because these genes are directly involved in successful parasitism, they naturally constitute interesting targets to develop new control measures. Provided that these proteins are specific to parasitic species they can lead to the development of more targeted and specific control measures. In an *a priori*-based approach, we searched within protein set 1, those presenting the same characteristics than known effectors. Typically, effector proteins bear a signal peptide for secretion and no transmembrane region. We also had identified previously, using the MERCI software [14], a set of protein motifs that are frequent in known effector proteins but absent from housekeeping proteins in RKN. Among protein set 1, we found a total of 3,311 proteins that possessed a signal peptide, 2,453 that possessed an effector MERCI motif and 13,856 that had no predicted transmembrane region. Overall, out of the 15,952 proteins present in set 1, we found 993 proteins that cumulated all these 3 criteria and thus have the same characteristics than canonical effectors.

### Transcriptional support of putative novel targets and further pruning

Because the *M. incognita* and *M. hapla* proteins have been deduced from the gene models predicted as part of automated genome annotations [6], [8], set 1 may contain a proportion of proteins deduced from wrongly or over-predicted genes. To minimize the risk of functionally analyzing proteins representing false predictions, we required two additional criteria. (i) The protein must be present in at least two different plant-damaging organisms (including the two RKN species) and, (ii) the corresponding gene must be supported by transcriptomic data from RKN. We had previously assembled the ensemble of available *M. incognita* EST data, as described in [15]. This represented a total of 63,816 ESTs assembled in 22,350 distinct unisequences. Although substantial, this dataset can still be viewed as relatively limited. To complete this relatively scarce transcriptomic dataset, we generated RNA-seq transcriptome sequencing for six different developmental life stages of *M. incognita* (Table 1 and methods). RNA-seq generated more than 190 million reads in total that were assembled in 137,733 contigs (methods). Combined with available ESTs, this dataset is likely to encompass a significant proportion of the diversity of transcripts in a RKN. Out of the 15,952 proteins in set 1, a total of 5,530 had a corresponding CDS sequence that received significant transcriptional support from RKN ESTs or RNA-seq data (methods).

**Table 1:** Samples used for RNA-seq and resulting contigs.

Life stage	Reads	Reads used for assembly*	Contigs
Eggs	25,958,384	12,467,657	26,570
Early sedentary	29,278,684	13,295,793	18,485
Parasitic sedentary	33,750,791	15,558,421	18,045
Stage 3 and 4 larvae	25,126,758	12,859,902	8,272
Adult female	26,177,405	14,953,875	13,071
Adult male	25,464,740	16,995,332	29,321
Mixed stages	24,901,111	12,082,564	23,969

* after trimming, collapsing and cleaning as described in methods.

From the set of 109 putative transcription factors identified during the functional annotation, a total of 12 were supported by expression data and were present in at least two plant-damaging species (Figure 1).

From the set of 993 effector-like proteins, 232 were present in at least two plant-damaging species and were transcriptionally supported by alignments with Meloidogyne ESTs or RNA-seq data (Figure 1, Table S2). Among these 232 effector-like proteins, we found 42 previously reported RKN effectors, including SXP/RAL-2 like proteins [16], Venom Allergen-like Proteins (VAP) [17], Chorismate mutases [18], Cathepsin L-like protease 1 (MiCpl1) [19] as well as 32 plant cell wall-degrading enzymes, encompassing cellulases, xylanases, pectate lyases and expansin-like proteins [20]. Finding previously known and characterized Meloidogyne effectors among our list of predicted effectors constituted an important validation of our approach. Because the main aim of our genome mining approach was to find novel potential targets we were exclusively interested in the 190 remaining effector-like proteins. Out of these 190 novel effector-like proteins, only 25 different Pfam domains were found in 46 proteins. Because they all received transcriptional support from *M. incognita* and have a homolog in at least one additional plant-damaging species, we can rule out the hypothesis that they are the product of over-prediction due to gene calling software.

### Experimental validation of targets by *M. incognita* infestation on tomato plants after gene silencing

Having identified novel putative transcription factors and effector-like proteins, present in plant-damaging species but absent from blacklisted ones, we wanted to experimentally validate their potential as amenable targets for the development of new control methods. Basically, we targeted selected genes one by one using small interfering RNAs (siRNA) on *M. incognita* infective J2 larvae, and infected host tomato plants with treated larvae. Six weeks after inoculation, we compared the numbers of galls and egg masses in siRNA-treated and control nematodes, as described in the methods.

Starting from the 12 putative transcription factors and 190 novel effector-like RKN proteins, we further pruned the list according to the following criteria. Because we perform biological assays on *M. incognita*, we first discarded proteins from *M. hapla* that had no ortholog in *M. incognita*. To avoid potential compensation of the silencing effect by gene copies performing similar function, we also removed all proteins that were encoded by multigene families. We ended up with a list comprising one putative transcription factor and 39 non-redundant effector-like proteins found in *M. incognita*, present in at least one other plant-damaging species, transcriptionally supported and without a homolog in a blacklisted species (Figure 1). We examined the corresponding coding sequences for compatibility with the design of specifically-matching siRNAs and the design of quantitative PCR primers (methods). We were able to design specific siRNA as well as specific PCR primers for the putative transcription factor (Minc07817) as well as for 15 out of the 39 genes encoding effector-like proteins. These 16 protein-coding genes were all present both in the *M. incognita* and *M. hapla* genomes. A total of 13 of the corresponding proteins do not have any predicted Pfam-A domain and, hence, no indication of the potential molecular function they may be involved in is available. One of the proteins (Minc03866) had a predicted C-type lectin domain and another (Minc03313) had an Astacin (peptidase family M12A) domain.

#### Effect of siRNA on the 16 novel target genes

To test whether the designed siRNA interfere with the expression of each of the 16 novel target genes, we performed Real-Time quantitative PCR (qPCR) experiments on soaked J2s (methods).

**Figure 5:**
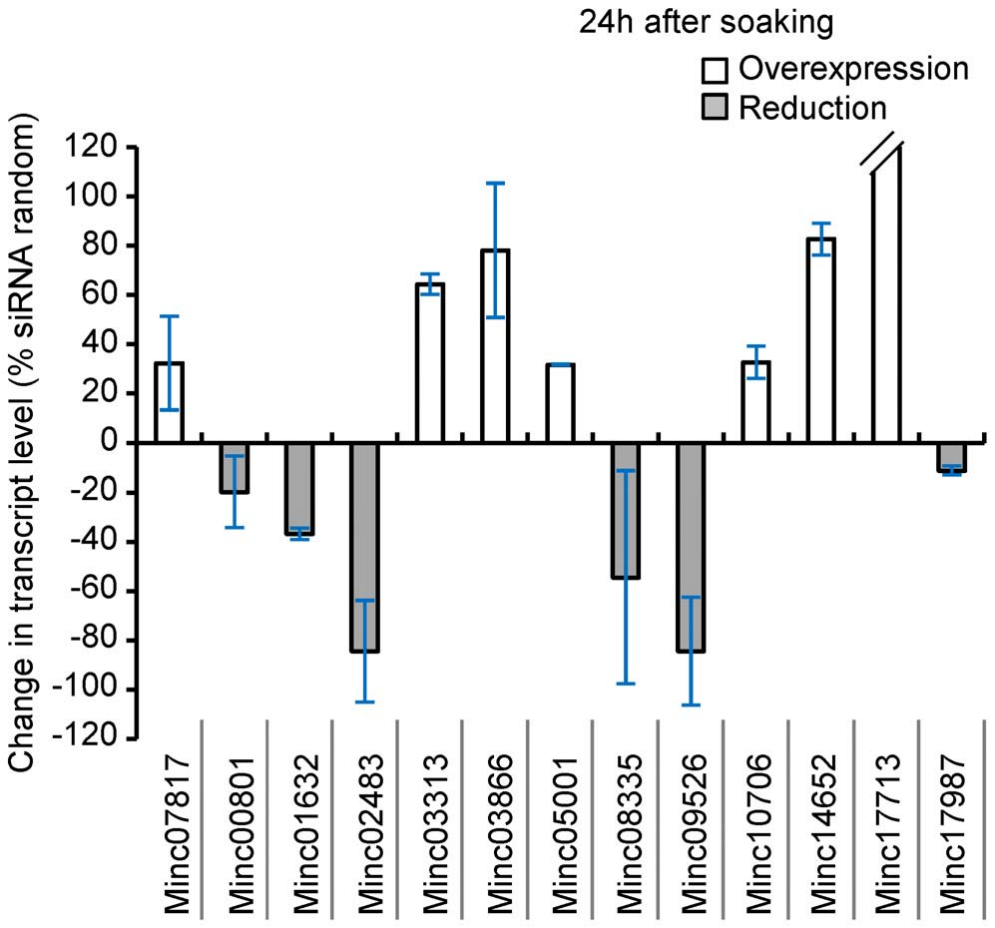
Transcript abundance percentage change in siRNA soaked J2s relative to control. siRNAs induced significant change in the targeted transcripts expression level. Transcript level for each of the targeted gene was measured by qPCR 24*M. incognita* genome (siRNA random).

Six siRNAs induced significant reduction in the corresponding targeted transcripts (Minc00801, Minc01632, Minc02483, Minc08335, Minc09526, Minc17987) 24 h after soaking treatment, compared to their expression level in control samples (Table 2, Figure 5). On the other hand, Minc08013, Minc08014 and Minc12224 siRNAs induced a diminution that was not reproducible between independent qPCR replicate experiments (data not shown). Surprisingly, 7 siRNAs (Minc03313, Minc03866, Minc05001, Minc10706, Minc14652, Minc17713, Minc07817) induced significant and reproducible increase in transcript abundance when qPCR was performed 24 h after soaking. A similar ‘bounce’ effect, that could be due to a response of the nematode RNAi machinery to increased siRNA quantities within the cells, had already been reported in PPN after gene silencing [21], [22], [23]. To test this ‘bounce’ effect, we performed qPCR analysis 16 h after soaking on Minc07817, Minc03866 and Minc05001. Confirming siRNA knockdown and bounce effect, Minc03866 was significantly silenced 16 h after soaking. However, Minc05001 expression was reduced but not significantly and Minc07817 expression was still higher than control (Figure S1). Overall, our qPCR results indicate that the designed siRNAs, were efficient to knockdown 7 out of the 16 target genes at the tested time points.

**Table 2:** Results of infestation, qPCR, and *in-situ* hybridization.

Gene	Trans. level variation	Reduct. of infestation*	Gall # reduction*	Egg mass # reduction*	ISH localization
Minc00801	Down @24 h	Yes	Not repro	Yes	Intestinal
Minc01632	Down @24 h	Yes	Yes	Yes	Ubiquitous
Minc02483	Down @24 h	Yes	Yes	Yes	Nerve ring
Minc03313	Up @24 h	Yes	Not repro	Yes	No signal
Minc03866	Up @24 h, Down @16 h	Yes	Yes	Yes	Subv. gland cells
Minc05001	Up @24 h, Not repro @16 h	Yes	Yes	Yes	No signal
Minc07817	Up @24 h, Up @16 h	Yes	Not repro	Yes	No signal
Minc08013	Not repro	Yes	Yes	Not repro	No signal
Minc08014	Not repro	Not repro	Not repro	Not repro.	Unchecked
Minc08335	Down @24 h	Yes	Not repro	Yes	Ubiquitous
Minc09526	Down @24 h	Yes	Yes	Yes	No signal
Minc10706	Up @24 h	Not repro.	Not repro	Not repro.	Unchecked
Minc12224	Not repro	Yes	Not sign.	Yes	Ubiquitous
Minc14652	Up @24 h	Not repro	Not repro	Not repro.	Unchecked
Minc17713	Up @24 h	Yes	Not repro	Yes	Intestin
Minc17987	Down @24 h	Not repro	Not repro	Not sign.	Unchecked

* statistically significant and reproducible reduction.

#### Effect of siRNA soaking on nematode infestation

For each of the 16 newly identified targets, we tested whether nematodes soaked with matching siRNAs showed a significant reduction in the numbers of galls and/or egg masses on infected plants as compared to control nematodes (methods).

**Figure 6:**
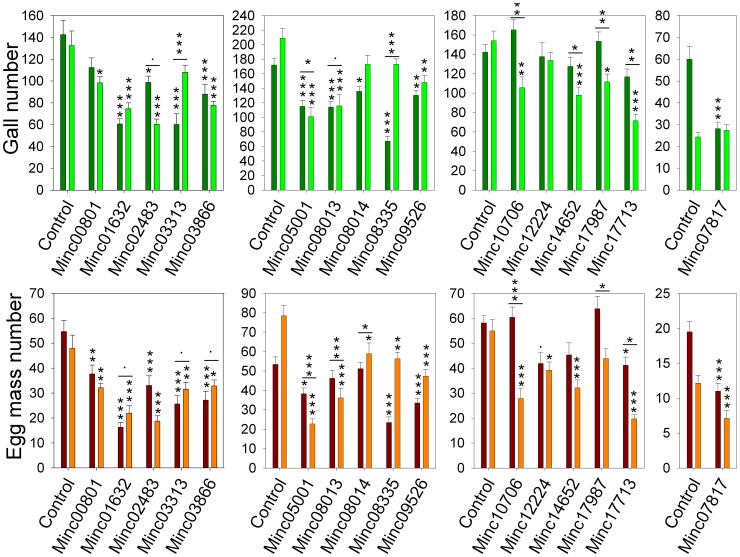
Effect of siRNA on nematode infestation. Controls are J2 larvae soaked with siRNA targeting no sequence in the *M. incognita* genome, accession numbers indicate M. incognita genes targeted by siRNAs. Infection tests were performed in duplicate represented as dark color bars for the first replicate and light color bars for the second replicate. Variations of number of galls and of numbers of egg masses, measured six weeks after inoculation, are represented in the top panels in green and in the bottom panel in red/orange, respectively. Error bars were calculated by standard error of the mean (SEM). P-Value above each bar indicates measures statistically different from controls. A significant change between the two duplicates is represented by p-values above a line spanning duplicates. P-value signification codes are as follows: 0.0001 ‘***’, 0.001 ‘**’, 0.01 ‘*’, 0.05 ‘.’.

Overall, 12 out of the 16 siRNA-treated samples (Minc00801, Minc01632, Minc02483, Minc03313, Minc03866, Minc05001, Minc08013, Minc08335, Minc09526, Minc12224, Minc17713 and Minc07817) showed a significant and reproducible reduction in the number of galls or egg masses after nematode infestation compared to control nematodes (Table 2, Figure 6, Table S3).

Genes that presented the highest reproducible reduction in number of galls were Minc01632 (43.67% to 57.48%), Minc03866 (38.36% to 41.20%), Minc05001 (33.03% to 51.54%) and Minc08013 (33.03% to 51.54%). Genes presenting the most important reproducible effects on the reduction of egg mass numbers were Minc01632 (54.17% to 70.09%), Minc02483 (39.54% to 60.85%, and Minc09526 (37.08% to 39.74%). A total of 5 targeted genes (Minc01632, Minc02483, Minc03866, Minc05001, Minc09526) showed significant and reproducible reductions of both the number of galls and egg masses (Table 2).

To check whether the observed effect on plant infestation was due to a toxicity of the siRNA treatment itself, we measured the viability and motility of the nematodes 1 hour and 16 hours after siRNA treatment. Comparison with control nematodes revealed that there was no significant and replicable toxicity effect (Figure S2).

#### *In situ* hybridizations of the 16 novel candidate targets

Among the 16 siRNA experiments (15 on effector-like genes and one on the putative transcription factor), 12 yielded significant and reproducible reduction of the number of galls or egg masses. Because all these genes are novel candidates and nothing is known about their possible functions, we investigated whether information could be gained from their expression localization using *in-situ* hybridization (methods). Results of *in-situ* hybridizations could be grouped in 5 different categories (Figure S3, Table 2). (i) Genes with ubiquitous expression detected (Minc01632, Minc08335, Minc12224). (ii) Genes with expression detected specifically in secretory subventral gland cells (Minc03866). (iii) Genes with expression detected specifically in the intestinal tract (Minc00801, Minc17713). (iv) Genes with expression detected specifically in the circumpharyngeal nerve ring (CNR), a structure surrounding the metacorpus, a pump-like organ (Minc02483). (v) Genes that returned no detectable signal (Minc03313, Minc05001, Minc08013, Minc09526, Minc07817).

#### Putative expression pattern of the 16 novel candidate targets

Because we have generated RNA-seq data for 6 developmental life stages in *M. incognita*, information on the genes expression pattern can be obtained in addition to raw transcriptional support. Aligning these RNA-seq reads separately for each life stage on the *M. incognita* genome (methods) allowed estimating the relative expression level of the 16 novel candidate targets in the 6 developmental life stages (Table S4). Interestingly, although there was no evident common expression pattern, all 16 genes were expressed at a substantially high level in at least one parasitic stage (early sedentary, parasitic sedentary, J3 and J4 larvae and adult female). This is consistent with the measured effect on infestation following soaking of siRNA at the infective J2 larvae stage.

## Discussion

### Orphan genes and genes shared with other plant-damaging species

Out of the 34,780 predicted proteins from the *M. incognita* and *M. hapla* whole proteomes, we have eliminated a total of 15,181 proteins because they had predicted orthologs in at least one of the 18 blacklisted species, based on OrthoMCL. In comparison, our taxonomic BLASTp analysis against the NCBI's nr library allowed elimination of only 1,201 further RKN sequences. This result suggests that our OrthoMCL filtering was able to eliminate most of the RKN proteins having potential orthologs in non-target species. Despite our selection of 23 species compared to the RKN is far from representing a significant portion of the whole biodiversity available, it constituted a stringent filter, probably because representatives from various different lineages, ranging from fungi to vertebrates, were included. This OrthoMCL filter also allowed us to dramatically reduce the number of proteins to be compared with the nr library in subsequent BLASTp comparison. The 1,201 sequences eliminated at the taxonomic BLASTp step probably consisted of gene families not represented among the 23 compared species. Besides allowing elimination of proteins having orthologs in non-target species, the OrthoMCL and BLASTp filters also allowed identification of RKN genes shared by several plant-damaging species. A total of 5,297 non redundant RKN proteins were present in at least two plant-damaging species but absent in non-target species, according to OrthoMCL and BLASTp filters. These proteins, apparently restricted to plant-damaging species, may be involved in core mechanisms common to several of these agricultural pests. Another point of interest revealed by the OrthoMCL and BLAST analyses is the set of potential orphan genes in RKN. A total of 5,536 non-redundant RKN proteins neither returned predicted orthologs in the OrthoMCL analysis nor had any significant BLASTp hits, in other species. These apparently RKN-restricted proteins can represent true orphans but may also be the result of possible artifacts due to over-predictions made by gene calling software in RKN genomes. However, 949 of the corresponding genes received transcriptional support from EST or RNA-seq data and are thus unlikely to be the results of over-predictions. Similarly, 2,416 of these orphan genes are present both in the *M. incognita* and *M. hapla* genomes and it appears improbable that these genes have been over-predicted twice independently in two distinct genomes using distinct gene calling strategies. These genes, apparently restricted to RKN and otherwise orphan, may be involved in processes specific to RKN such as the fine interactions between the nematode and the plant host (e.g. induction of a feeding site in the plant) or in the ontogeny of specialized organs (e.g. gland cells or protrusible stylet).

Not only are those genes candidate targets for new treatments against RKN, but also fundamental genes to better understand adaptation to a plant-parasitic life.

Whether these genes are true orphans can be questioned when considering the relative scarcity of omics data available for plant-parasitic nematodes in general. Our OrthoMCL analysis included only two proteomes of plant-parasitic nematode species (*M. incognita* and *M. hapla*) and to date, no whole proteome for a phytoparasitic nematode species is present in the NCBI's nr database. Hence, these genes may have orthologs in other plant-parasitic nematode species. Availability of further whole genomes, transcriptomes and deduced proteomes from additional phytoparasitic nematodes in the future will allow us to decipher whether some of these genes are shared with other plant-parasitic species and may, consequently, be involved in core processes linked to this lifestyle.

### Nature of filters and novel candidate target genes

The series of filters we have set up in our bioinformatics pipeline resulted in a very stringent screening of the two whole RKN proteomes. We have first eliminated all proteins that had potential orthologs in a series of blacklisted species that must be preserved if new nematode control means, targeting these genes, were developed. We next ran two strategies in parallel to identify novel candidates in RKN proteomes that would be more clearly amenable for development of new control methods.

The first strategy was an *ab initio* data-driven one. Because we noticed an over-abundance of putative transcription factors in the set of RKN proteins absent from blacklisted species, we focused on this category. We identified 12 putative transcription factors absent from blacklisted species and supported by transcriptional evidence. If these proteins actually function as transcription factors, they may be involved in regulation of genes involved in RKN-specific functions such as parasitism genes or modulate the expression of host plant genes. One of those putative transcription factors was present as a single copy gene in *M. incognita* and was compatible with the design of specific siRNA and qPCR primers and thus amenable for biological assays.

The second strategy we used was an *a priori* based one. Because effector proteins secreted by nematodes are known to be important in their plant-parasitic ability, we searched proteins that featured the same characteristics and identified a list of 232 putative effectors. Validating our *a priori* strategy, we retrieved 42 proteins that were previously described as known effectors in the literature. Obviously, not all effectors previously described so far were found. This is mainly for the following reasons: (i) several known effectors do not possess an N-terminal signal peptide and/or a MERCI effector-motif (ii) some nematode effectors have homologs in blacklisted species. Because we were mainly interested in the discovery of novel potential targets, we focused our analysis on the 190 remaining novel effector-like proteins not present in blacklisted species. A total of 39 corresponding genes were not redundant in *M. incognita* and present in at least one other plant-damaging species. Out of these 39 genes, 15 were compatible with the design of siRNAs and qPCR primers and thus amenable for further biological assays.

During infestation tests on tomato plants, out of the 16 novel candidates identified (15 effector like and 1 putative transcription factor), 12 turned out to show significant and reproducible reduction in the number of egg masses or galls when treated with anti-candidate siRNAs.

Overall, our strategy was not to build a comprehensive list of candidate genes that might produce the most severe phenotypes on nematodes. In contrast, the originality of our approach was to focus from the beginning on genes that were present in plant-damaging species but absent from non-target “blacklisted” species. We thus produced a stringent and restrictive list of candidates that cumulated a series of characteristics that made them the most promising candidates for the development of safer and more-specific control methods.

### Infestation assays

Overall, after siRNA soaking, we measured significant and reproducible effect on infection on 12 targeted genes. This effect was measured by a diminution in the number of galls or egg masses. Reduction in the number of galls implies that fewer nematodes have managed to induce a feeding structure. A reduction in the number of egg masses signifies that fewer female nematodes have managed to complete their development until the production of egg masses, a necessary step to propagate the infection at the next generation. In 6 cases, we measured a reproducible and significant reduction in the number of galls. Interestingly, 5 out of these 6 cases also led to significant and reproducible diminution of the number of egg masses. This observation makes sense since reduction in the capacity of nematodes to form galls will have direct downstream impact on the number of egg masses produced. Interestingly, targeting gene Minc01632 was responsible for both the most important reproducible diminution of the number of galls and of the number of egg masses. The corresponding protein is 155 amino-acids long and has neither significant similarity in the NCBI's nr database nor predicted protein domain, as most of the 16 identified novel targets.

In contrast, observing significant reduction of the number of egg masses does not necessarily require upstream reduction of the number of galls. Indeed, if the siRNA-targeted gene has functional consequences in processes that take place between the formation of galls and the production or extrusion of eggs we should observe a significant reduction in the number of egg masses but not in the number of galls. This is indeed what we observed for 6 targeted genes (Minc00801, Minc03313, Minc08335, Minc12224, Minc17713 and Minc07817). While reduction of the number of egg masses was significant and reproducible; reduction in number of galls was either not reproducible or did not reach the significance threshold.

Overall, we observed no correlation between reduction of infestation and a measurable effect on nematode motility or viability. This indicated that the effect on infestation was globally not due to a toxicity of the siRNA treatment. For instance, genes that showed among the most important reduction in the numbers of egg masses or galls (e.g. Minc01632>40% reduction or Minc09526∼40% reduction) did not show substantial diminution of viability or mobility 1 h or 16 h after soaking. We can thus deduce that the reduced infestation observed is generally not a consequence of reduced motility but more likely results from modification in other processes important for parasitism. Because the genes we have targeted are mostly specific to RKN and not shared by many species, we expected no systematic effect on viability or motility as opposed to evolutionarily conserved housekeeping genes [24].

### Effect of RNA interference on transcripts levels

Treatments with siRNAs had reproducible significant effects on target transcript levels in 13 out of the 16 samples tested (Table 2). Twenty-four hours after soaking, six genes showed a diminution of the transcript abundance while 7 yielded an increase of transcript level. Because we suspected a possible bounce effect, we randomly picked 3 of these 7 genes and measured transcript abundance at an earlier time point (16 h). One of the tested genes (Minc03866) showed a significant and reproducible diminution of transcripts level at this time point. It is possible that some of the six other genes that showed an increase of transcripts level at 24 h may also present an initial decrease at an earlier time point. Such bounce phenomenon has already been described in plant-parasitic nematodes [21], [22], [23]. Interestingly, the 13 siRNAs yielding effects on transcript level encompass 10 out of the 12 cases of reproducible significant reduction of infestation. Furthermore, for 7 genes (Minc00801, Minc01632, Minc02483, Minc03866, Minc08335, Minc09526, and Minc07817), following the siRNA treatment, there is both a significant and reproducible diminution of the abundance of transcripts and of the infestation of nematodes. Intriguingly, for two genes, there is significant and reproducible diminution of infestation but no significant effect on transcripts level. Investigating earlier or later time points may reveal significant effects. Alternatively, the corresponding mRNA may be sequestered away from the translation machinery without being itself degraded. Such a mechanism of translation repression without mRNA degradation has already been documented in plants and animals [25].

### *In situ* hybridizations

We performed *in situ* hybridization assays on the 12 genes that yielded significant reduction of infestation to try to gain information on their putative functions. Because 11 of the 12 tested genes share characteristics with known RKN effectors, it could be expected that they show transcription localization patterns similar to the known effectors. Canonical effectors are transcribed in secretory gland cells for injection by the nematode in plant tissue. We found one gene expressed specifically in the subventral gland cell (Minc03866). This gene could well encode an effector protein eventually secreted in plant tissue during infestation. Interestingly, when targeted *via* siRNA, this gene returned one of the strongest effect on reduction of infestation. Ubiquitous expression, which includes the secretory gland cells, was observed for 3 genes and these genes could be multi-functional, including possibly effectors depending on whether they are eventually secreted *in planta* or not. A total of 5 genes returned no detectable signal and although they may function as effector, there is no further supporting data from *in situ* hybridization assays. For the three other genes, expression localization does not support a possible secretion *in planta*, at least at the observed J2 stage. One gene (Minc02483) shows an expression localization specifically on nerve tissue surrounding a region called the metacorpus. The metacorpus acts as a pump to inject secretion or to take up nutrients from the nematode syringe-like stylet. It is possible that the gene expressed in the surrounding nerve cells may be involved in correct functioning of this pump. siRNA targeted against this gene led to the second strongest reduction effect on the number of galls and egg masses. The two other genes have an expression restricted to the intestinal tract and their targeting by siRNAs leads to significant and reproducible reduction of the number of egg masses. Lacking any known protein domain, it would be too speculative to predict any function for the corresponding gene products.

### Potential for the development of novel control methods

Using soaking experiments with siRNAs targeting each of the 16 identified novel genes, we noticed a significant and reproducible diminution of infestation in 12 cases. These results were obtained by inoculating infective J2 larvae after one hour soaking in a solution containing a siRNA concentration of 0.05 mg/ml. Although siRNA delivery *via* soaking can be relatively efficient because of systemic propagation of the RNA interference, levels of inactivation can vary and duration of the effect is poorly known [26]. Thus, it is possible that some of the genes we have identified would show significant reduction of infestation only when targeted at later stages of the nematode life cycle. Unfortunately, J2 infective larvae is the only free-living stage that can be targeted with soaking approaches, the rest of RKN life cycle takes place within plant tissue. However, delivery of siRNA at later stages can be imagined by development of transgenic plants expressing these interfering RNAs. Because the nematode feeds on root cells, siRNA can be actively delivered and it can be hypothesized that this mode of delivery is more efficient than passive soaking. Supporting this idea, genetically modified plants that express interfering RNAs have already proved to be efficient at reducing nematode infestation, at least in laboratory conditions (for review [26], [27]). Before such transgenic plants can possibly reach the market, their bio-safety must be assessed. Because the genes we have identified are absent from species concerned by bio-safety risk (including human and the host plant), development of such transgenic plants is a promising potential application towards novel control methods of RKN. Alternatively, and because genetically-modified plants are not well accepted, especially in Europe, agrochemical approaches to develop new compounds specifically targeting one or several of the genes we have identified can be considered. However, this probably represents a less evident and straightforward strategy.

### Conclusion

Overall, our bioinformatics RKN genome screen has led to the identification of a series of genes present in multiple plant-damaging species that probably play important roles in successful parasitic interactions. In the absence of genetic tools to test the effect of gene knock-out in RKN, we have opted for an RNAi gene knock-down strategy. Despite potential limitations in detecting physiological effects, siRNA treatment yielded significant and reproducible reduction of infestation in 12 out of the 16 testable cases. Overall, 5 siRNAs yielded both a diminution of egg masses and galls and this diminution was correlated with a diminution of the abundance of transcripts in the corresponding gene. These 5 genes probably represent the most promising targets for the development of novel efficient control means more specific and safe for the environment.

## Materials and Methods

### Bioinformatics

#### ORTHOMCL comparative analysis

We compared the whole protein sets of *M. incognita* and *M. hapla* against those of 23 other species using OrthoMCL [9] with default parameters to detect putative orthologs based on a reciprocal best blast hit approach. Criteria for species selection are explained in the results section. We used in house Perl scripts to extract RKN genes that had either no predicted orthologs in the 23 other selected species at all or orthologs only in parasites, phytopathogens or phytophagous species. To avoid redundancy between *M. incognita* and *M. hapla* proteins, we only kept *M. incognita* sequences as representative whenever proteins from both the two RKN were present in a same OrthoMCL cluster. All against all BLASTp analysis of the 25 protein sets have been performed on a computational grid (ProActive PACA grid: http://proactive.inria.fr/pacagrid/).

#### Taxonomic BLASTp analysis

BLASTp analyses were performed with an e-value threshold of 0.01 and without low-complexity filter against the NCBI's nr database at the protein level. BLASTp hits were considered as significant based on adapted percent identity and percent of alignment length thresholds. These thresholds were determined by examining the average lowest percent identity and query length coverage from one-to-one orthologs between RKN and the other species obtained during the OrthoMCL analysis (Table S5). We distinguished two different cases depending on whether or not the subject species is a Metazoan. For Metazoan subjects, BLASTp hits were considered as significant if they aligned with at least 40% identity on at least 70% of the RKN query protein length. For non-metazoan subjects, BLASTp hits were considered significant if they aligned with at least 30% identity on at least 50% of the query length.

Similarly to the OrthoMCL analysis, we eliminated every RKN protein that presented a significant BLASTp hit with at least one blacklisted species. For the BLASTp analysis, we blacklisted 4 different whole taxa, representing a total of 170,258 species:

- Chordata: 46,011 species
- Annelida: 4,551 species
- Mollusca: 11,932 species
- Viridiplantae: 107,764 species

All RKN proteins that did not return a significant hit in one of these species or that did not return any significant hit at all were kept for the rest of the analysis.

In parallel, we tagged RKN proteins that were not eliminated and returned a significant hit in at least one “plant-damaging species” as shared with another plant parasite.

#### Constitution of a database of “plant-damaging species”

We established a list of species that are known plant-pathogens, plant-parasites or known to feed specifically on plant material, using available databases dedicated to plant-interacting organisms as well as prior knowledge on phylogenetic clades containing plant-pathogens or plant-parasites.

We first retrieved the list of species present in the “Comprehensive Phytopathogen Genomics Resource” (CPGR) database [28]. This list contains 806 referenced species and strains, including 63 bacteria, 56 fungi, 16 nematodes, 12 oomycetes, 36 viroids and 623 viruses. For some species, several strains are listed and refer to the same NCBI's taxonomy identifiers (TaxIDs). In total, 794 distinct corresponding TaxIDs could be listed. Besides CPGR, we retrieved the list of species having plant as hosts in the “Pathogen Host Interaction database” (PHI-base) [29]. This allowed retrieval of 54 species, including 46 fungi, 3 bacteria and 5 oomycetes. Elimination of redundancy between the lists of species extracted from the CPGR and from PHI-base led to a total of 788 different species (distinct TaxIDs), including 63 bacteria, 50 fungi, 14 nematodes, 8 oomycetes, 619 viruses and 34 viroids.

Because we aimed at producing the most comprehensive possible list of plant-damaging species, we completed the non-redundant list extracted from CPGR and PHI-base with whole nodes from the NCBI's tree of life:

- Plant-parasitic nematodes, 4 nodes: Tylenchida (879 species, e.g. root-knot nematodes, cyst nematodes etc.), Nordiidae (26 species, e.g. *Pungentus thornei*, *Longidorella parva* etc.), Longidoridae (130 species, e.g. *Xiphinema index*, *Longidorus sylphus* etc.) Trichodoridea (30 species, e.g. *Trichodorus primitivus*, *Paratrichodorus minor*).
- Plant-parasitic insects, 1 node: Aphididae (448 species, e.g. *Acyrthosiphon pisum*) and phytophagous insects (mostly), 1 node Lepidoptera (24,551 species, e.g. *Spodoptera frugiperda*).
- Plant-pathogenic Oomycetes, 4 nodes: Pythium (484 species, e.g. *Pythium ultimum*), Phytophtora (363 species, e.g. *Phytophthora ramorum*), Peronosporaceae (312 species, e.g. *Hyaloperonospora arabidopsidis*), Albugo (17 species, e.g. *Albugo candida*)+2 species: *Aphanomyces cochlioides*, *Aphanomyces euteiches*.
- Plant-parasitic Trypanosomatidae, 1 node: Phytomonas (82 species, e.g. *Phytomonas serpens*).

Overall, the list we have constituted contains 834 distinct TaxIDs (species and nodes), including 18 from nematodes, 16 from oomycetes, 79 from fungi, 65 from bacteria, 1 from trypanosomatida, 2 from insects, 619 from viruses and 34 from viroids. This represents a total of 28,054 plant-damaging species.

#### *In silico* functional annotation

Known protein domains in the 34,780 RKN proteins were searched using HMMER 3.0 [30] against the Pfam-A database of manually curated HMM profiles [31]. Corresponding Gene Ontology (GO) terms assigned to Pfam domains have been retrieved using a Perl script developed for this occasion. For comparative purpose between the two RKN proteomes and between the set of RKN proteins that passed both the OrthoMCL and taxonomic Blast filters (protein set 1), we mapped all the G.O. terms to the generic GO-slim ontology containing only parent terms. Consequently, the different datasets are all annotated at the same granularity level which allows direct comparisons. To map the general GO terms to the GO-slim ontology, we used the Perl module GO-Perl and the Perl script map2slim. We used a Fisher's exact test to assess whether observed differences in relative abundances of GO terms were statistically significant.

#### Searching effector-like proteins

Signal peptides for secretion were searched using SignalP 3.0 [32] with both neural network and hmm based methods. Whether predicted signal peptides were supported by one or both methods was reported in a dedicated database. We also searched transmembrane regions using TMHMM [33] with default parameters and stored the results in our database. Domains specifically frequent in known RKN effectors, previously identified using the MERCI software [14] were searched in the two whole RKN protein sets.

#### Transcriptional support

To assess whether a gene was supported by transcriptional data, we used several different sources of evidence. Basically, we aligned the coding sequences (CDS) corresponding to the RKN proteins to different transcriptomic libraries using BLASTn. We aligned the CDS to 22,350 distinct *M. incognita* EST contigs generated as described in [15] and to 137,733 contigs generated from the RNA-seq transcriptome sequencing of 6 different *M. incognita* developmental life-stages as described below. Both for EST or RNA-seq contigs, we set the e-value threshold to 1e-20. To have comparable bit scores and e-values between the different sequence libraries, we manually set the size of the database to z = 60,000,000 sequences. We considered a CDS as transcriptionally supported, provided that it returned alignments with at least 98% identity on at least 80% of the EST or RNA-seq contig length.

#### Processing, mapping and assembly of RNA-seq data

All the sequence libraries were assembled *de novo* using Velvet/Oases software after elimination of reads of low quality. Adaptators were removed and reads redundancy at 100% identity level was eliminated (collapsing) before assembly. Reads longer than 25 bp were assembled with velvet_1.0.15/oases_0.1.18. Number of predicted contigs (i.e. transcripts) ranged from ∼8,500 for *M. incognita* parasitic stage to ∼30,000 for adult males. Assembled reads for the different developmental life stages are available for download at the following URL: http://www7.inra.fr/meloidogyne_incognita/genomic_resources/downloads. Individually for the 6 developmental life stages, cleaned reads were aligned to the *M. incognita* genome using Bowtie2 [34] and Tophat2 [35]. Gene expression patterns were deduced from the aligned reads, using Cufflinks [36], according to the protocol published in [37] and presented as RPKM values in Table S4.

### Biological experiments

#### Sample preparation and RNA-seq Illumina sequencing

A total of 6 different life stage samples (Table 1) were collected from tomato roots (*Solanum esculentum* cv. St Pierre) by incubation in 10% (v/v) Pectinex (Novozymes, Bagsvaerd, Denmark) and 5% (v/v) Celluclast BG (Realco, Louvain-la-neuve, Belgium) for 3 hours, respectively 10, 40 and 60 days after inoculation. Males were collected as previously described [38], [39]. All the other samples were purified from root debris by sucrose gradient centrifugations. RNA isolation using TRIzol Reagents (Invitrogen, Carlsbad, CA, USA) was done according to protocol available from Invitrogen and re-suspended in 10 µl RNase-free water. Purity and concentration of the RNA was determined on a 2100 Bioanalyzer (Agilent Technologies, Santa Clara, CA, USA) and cDNA was only produced from high RNA quality (RIN>7). Reverse transcription was carried out using the Ovation pico WTA System (NuGEN Technologies, Inc, San Carlos, CA, USA).

The cDNAs were sonicated separately to a 150- to 600-bp size range using the S2 covaris instrument (Covaris, Inc., USA). Single end libraries were prepared following Illumina protocol (Illumina DNA sample kit). Briefly, fragments were end-repaired, then 3′-adenylated, and Illumina adapters were added. Ligation products of 350–400 bp were gel-purified and size-selected DNA fragments were PCR-amplified using Illumina adapter-specific primers. Libraries were purified and then quantified using a Qubit Fluorometer (Life technologies) and libraries profiles were evaluated using an Agilent 2100 bioanalyzer (Agilent Technologies, USA). Each library was sequenced using 76 base-length read chemistry in a single flow cell on the Illumina GA IIx (Illumina, USA).

#### siRNA design and siRNA treatment for viability tests and infection assays

siRNAs (Figure S4) were prepared using the Silencer siRNA Construction Kit (AM1620, Ambion, Austin, TX) and siRNA yields were determined using a NanoDrop 2000 spectrophotometer (NanoDrop Products, Wilmington, DE). For control, we used a siRNA designed to have no sequence similarity in the *M. incognita* genome [40] as confirmed by BLASTn searches. siRNAs were conserved at −80°C in 2 µg aliquots until use. Eggs of *M. incognita* were collected from tomato plants (*Solanum esculentum* cv. St Pierre) cultured in greenhouse. Eggs were collected as described by Rosso *et al.* [38] and J2s were hatched in water. About 10,000 J2s were soaked in 40 µl final volume of spring water in the presence of 0.05 mg/ml siRNA for 1 hour. Worms were washed twice with water by centrifugation at 10,000 g for 1 min and suspended in 100 µl of water. For infection assays, roots of 24 tomato plants aged of four weeks were each inoculated with 250 *M. incognita* infective J2 larvae, previously washed and oxygenated over night in spring water as recently described [41]. Two replicates of the infection assays were performed at three weeks intervals. Galls and egg masses were counted six weeks after inoculation. Statistical analyses were performed by an ANOVA test using the R software. For viability/motility assay, J2s were also soaked with siRNAs for one hour. The number of dead J2s was counted under microscope observation on 100 individuals, one hour and 16 hours after soaking and washing. We also analyzed movement quality and rapidity for 25 individuals. For movement quality we observed extremity and whole length movement and the quality of movement was noticed by rapidity of undulation or absence of undulation.

#### Quantitative-PCR

RNA was isolated from approximately 500 J2s soaked for 1 hour in 0.05 mg/ml siRNA and incubated for 16 h or 24 h in water, using TRIzol Reagent (Invitrogen, Carlsbad, CA, USA). Purity and concentration of the RNA was determined on a NanoDrop_2000 spectrophotometer (NanoDrop Products, Wilmington, DE). Reverse transcription was carried out using the iScript cDNA Synthesis Kit (Bio-Rad laboratories, Marnes la Coquette, France). The primers for qPCR were determined using primer3 software [42] and synthesised by Eurogentec (Seraing, Belgium) (Figure S4). The cDNA was diluted 10 times, and 5 µL was used per PCR reaction. Seven and a half microliters of 2× SYBR Green Master Mix (Eurogentec, Liege, Belgium), 0.2 µL each of 1001µM of forward and reverse primer, and 4.61µL of water were added to the cDNA. Thermocycling was carried out with one cycle at 95°C for 15 min, followed by 40 cycles of 95°C for 15 sec and 56°C for 1 min and 72°C for 30 sec. The dissociation curve of the final products was checked to ascertain the presence of a single amplification product. qPCR was performed on triplicate samples of each cDNA. Among the three tested candidate reference genes, i.e. *M. incognita* polygalacturonase (*Mi-pg-1*, Minc18543), Glyceraldehyde 3-phosphate dehydrogenase (GAPDH, Minc10963) and Actin α (Minc06773), GAPDH was determined as the most stable reference using Genorm algorithm [43] (Data not shown) and was selected as reference gene. For normalization, the threshold cycle values of GAPDH amplifications (CT_GAPDH_) were subtracted from the threshold cycle values of the analyzed genes (CTexp). Transcript levels in arbitrary units (AU) were calculated with the formula: AU = 100 * 2^(CTexp – CTGAPDH)^. Figure 5 shows results from two independent replicates.

#### Transcript analyses by *in situ* hybridization

Sense and antisense probes were synthesized from each target gene with specific primers designed with Primer3 software [42] and synthesised by Eurogentec (Seraing, Belgium) (Figure S4). As controls, we used the polygalacturonase *Mi-pg-1* (Minc18543) gene for esophageal gland-specific labeling and the *GAPDH* Minc10963 gene for ubiquitous labeling. *In situ* hybridizations were conducted as described previously [44]. 10,000 J2s were hybridized with DIG-labeled specific probes at 40°C over night for each target transcript.

## Supporting Information

Figure S1 **Transcript level measured 16 h after soaking to test bounce effect.** Expression level was measured for Minc07817, Minc03866 and Minc05001 and compared to nematodes treated with control siRNA 16 h after soaking to test siRNA knock-down effect at earlier time point.
(PPTX)

Figure S2 **Effects of siRNAs on motility and viability of nematodes.** Controls are J2 larvae soaked with siRNA targeting no sequence in the *M. incognita* genome, accession numbers indicate *M. incognita* genes targeted by siRNAs. Viability was assessed 1 h (A) and 16 h (B) after soaking by counting the number of dead nematodes (black bars), the number of individuals with movements restricted to extremities (red bars), movements on the whole length of the body (dark pink bars) and fast undulations (light pink bars) under microscope observation. Three independent replicates were analyzed. Error bars represent standard error of the mean. Variability from one replicate to another was generally too high and frequently higher than from one siRNA to another which precluded any statistical test from finding significant differences.
(TIF)

Figure S3 ***In situ*** **hybridizations by categories.** Localization of transcripts for the 12 genes that yielded significant and reproducible reduction of infestation. (I) Ubiquitous expression: Minc01632 Minc08335 and Minc1224. As a positive control, an antisense probe targeting NADPH transcripts was used. (II) Expression localized to subventral secretory gland cells: Minc03866. As a positive control, antisense probe targeted against Mi-PG1 transcript was used. (III) Expression localized to the intestinal tract: Minc00801, Minc17713. (IV) Expression is localized in the circumpharyngeal nerve ring: Minc02483. As a negative control, we used a sense probe designed on polygalacturonase gene Mi-PG1, known to be expressed in subventral gland cells. (V) No detectable signal.
(PPTX)

Figure S4 **Positions of qPCR primers and siRNAs on the 16 tested genes.** The positions of qPCR primers (forward in red, reverse in blue) as well as siRNAs (in green) are reported along the exon/intron structures of the 16 genes used for infestation assays and transcript level analyzes. The scale bars above each gene model represents a length of 100 nucleotides.
(PPTX)

Table S1 **Gene Ontology terms in** ***M. incognita*** **,** ***M. hapla*** **and in protein set 1.** For the 3 ontologies, (A) ‘biological process’, (B) ‘molecular function’, (C) ‘cellular components of the gene ontology, we report the abundance of GO-slim general ontology terms in *M. hapla*, *M. incognita* and protein set 1. Raw abundance and proportion of total terms in a given ontology for a given set are indicated. The last raw represents the difference in proportion between protein set 1 and the average proportion in the whole RKN proteomes. A color gradient from enriched terms in red toward depleted terms in green accompanies the values.
(XLSX)

Table S2 **232 effector-like proteins supported by transcriptional data.** We list the 232 effector-like proteins that received transcriptional support and associated information. We include, the RKN species (*M. hapla* or *M. incognita*), the protein accession number, the length in amino-acids of the corresponding protein, the presence in plant-damaging species according to OrthoMCL and BLASTp analyses, the source of transcriptional support, presence of a known Pfam protein domain, and, whether the protein is a known effector.
(XLS)

Table S3 **Percent reduction in number of egg masses and galls after siRNA treatment.** For the 16 genes tested with siRNA treatment, we report the percent reduction of the number of egg masses or of the number of galls. We list gene accession numbers and the category (effector-like or putative transcription factor). Values of percent reduction in the numbers of galls or egg masses compared to control are accompanied with standard error of the mean (SEM) values and a significance code for the associated p-value.
(XLS)

Table S4 **Expression pattern of the 16 novel candidate targets according to RNA-seq data.** The expression level for the 16 novel candidate target genes in each of the 6 developmental life stages (from left to right: eggs to adults) according to RNA-seq data. Values correspond to RPKM (reads per kilobase per million mapped reads) obtained by aligning RNA-seq cleaned reads to the *M. incognita* genome. A color gradient from red to green indicate low to high RPKM values for each gene (row), individually.
(XLSX)

Table S5 **Percent identity/length OrthoMCL.** Average percent identity and query protein length of 1-to-1 orthologs between species used in the OrthoMCL analysis are indicated. We separated the dataset into two pools (i) closely related (metazoan) species, (ii) distantly related (non-metazoan) species. Lowest obtained values are represented in red.
(XLS)
