# Supplementary figures and images for: Identification of Novel Target Genes for Safer and More Specific Control of Root-Knot Nematodes from a Pan-Genome Mining
Source: PLoS Pathog. 2013 Oct 31;9(10):e1003745. doi: 10.1371/journal.ppat.1003745 (PMC3814813; doi:10.1371/journal.ppat.1003745)

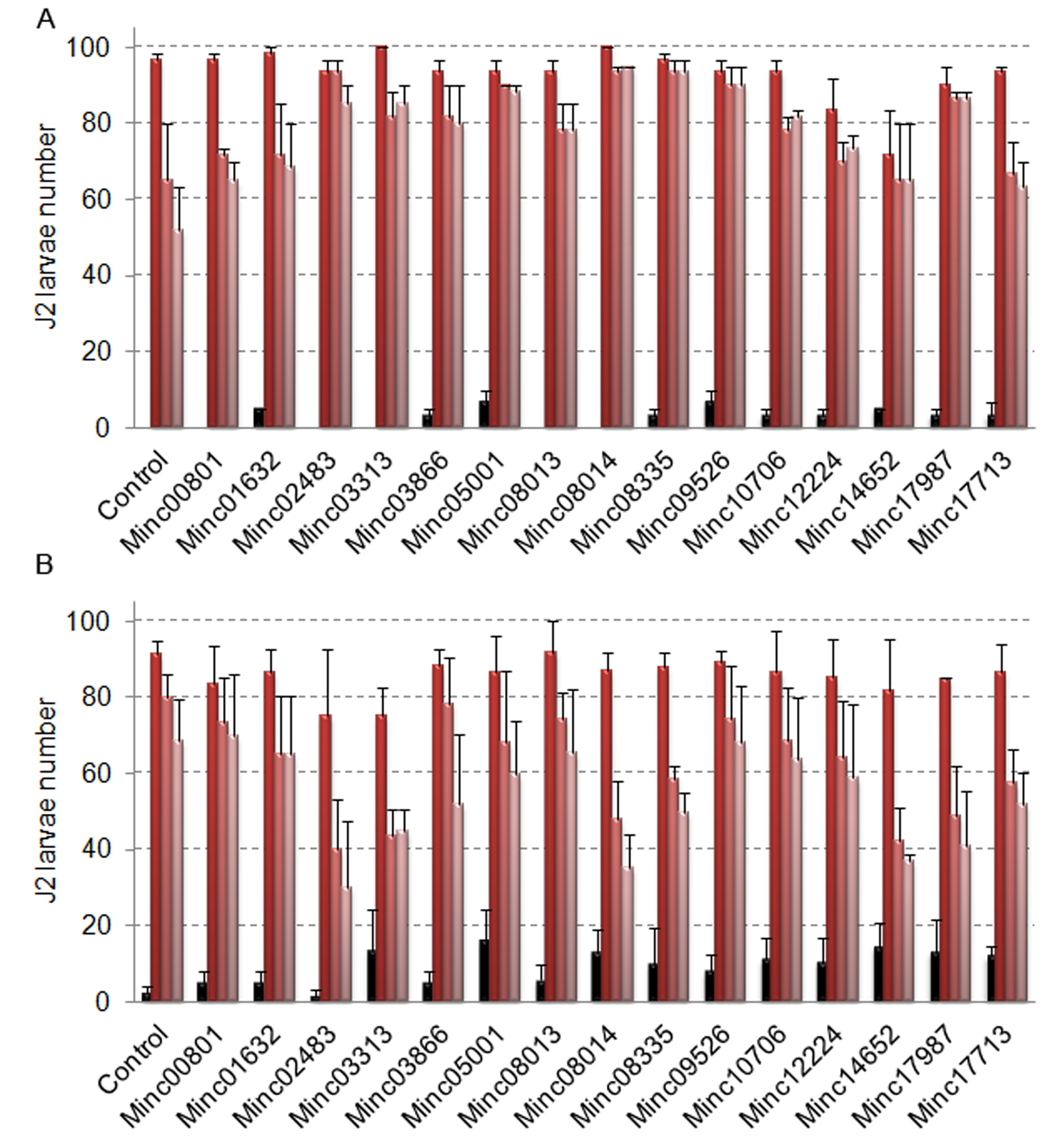

Supplement: Figure S2 — Effects of siRNAs on motility and viability of nematodes. Controls are J2 larvae soaked with siRNA targeting no sequence in the M. incognita genome, accession numbers indicate M. incognita genes targeted by siRNAs. Viability was assessed 1 h (A) and 16 h (B) after soaking by counting the number of dead nematodes (black bars), the number of individuals with movements restricted to extremities (red bars), movements on the whole length of the body (dark pink bars) and fast undulations (light pink bars) under microscope observation. Three independent replicates were analyzed. Error bars represent standard error of the mean. Variability from one replicate to another was generally too high and frequently higher than from one siRNA to another which precluded any statistical test from finding significant differences. (TIF) [file ppat.1003745.s002.tif]
